# Supplementary material for: Mutations in PCYT2 disrupt etherlipid biosynthesis and cause a complex hereditary spastic paraplegia
Source: Brain. 2019 Oct 22;142(11):3382–97. doi: 10.1093/brain/awz291 (PMC6821184; doi:10.1093/brain/awz291)
Supplement: awz291_Supplementary_Data [file awz291_supplementary_data.zip › awz291-Suppl_data/Supplementary_Data4.zip!/Supplementary information 4 - plasma lipidomics.zip!/Plasma lipidomics/Control vs Patient2/Statistical plots and data/Volcano plot_alternative.pdf]

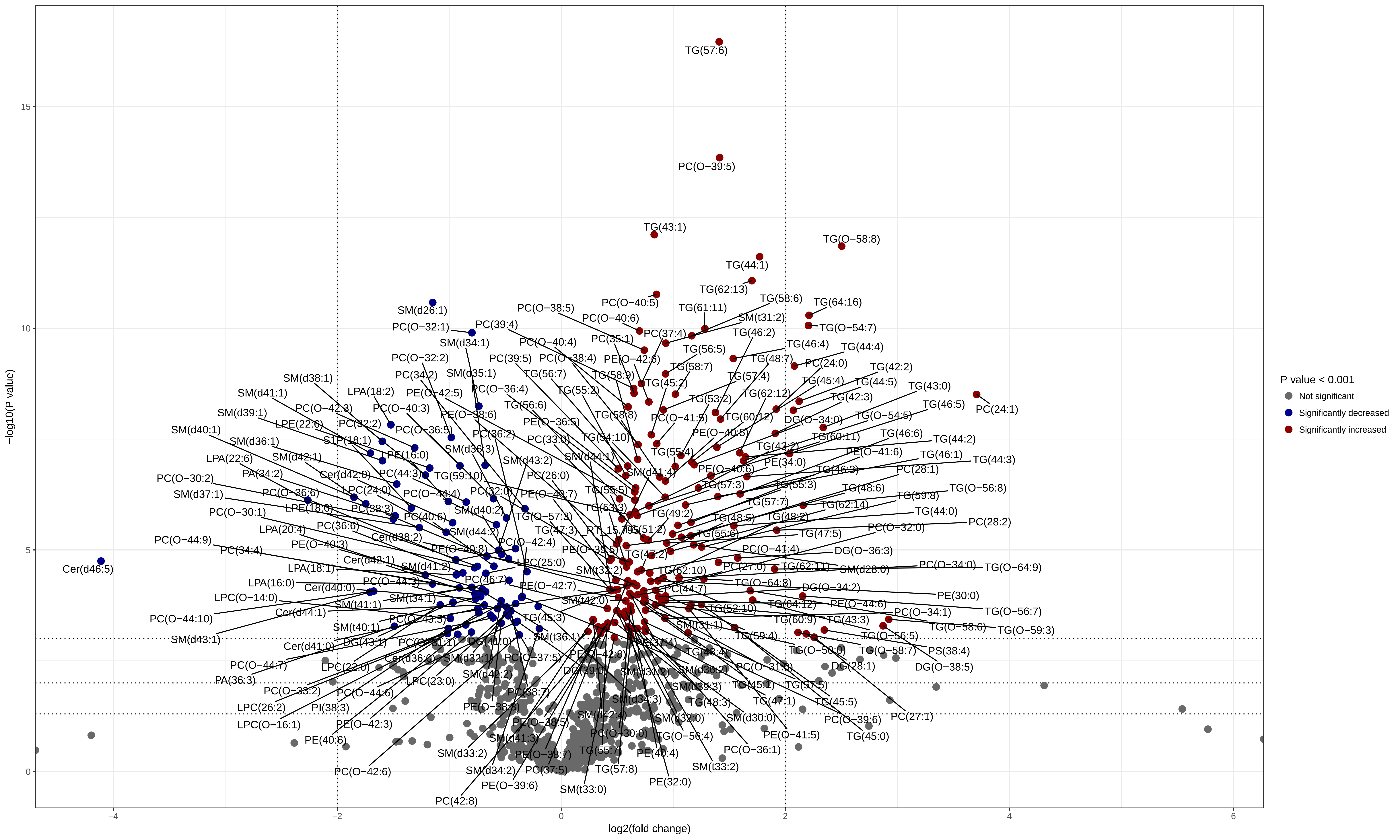
P value < 0.001
Not significant
Significantly decreased
Significantly increased
−log10(P value)
log2(fold change)
15
10
5
0
−4
−2
0
2
4
6
TG(57:6)
PC(O−39:5)
TG(43:1)
TG(O−58:8)
TG(44:1)
TG(62:13)
PC(O−40:5)
TG(58:6)
TG(64:16)
SM(d26:1)
PC(O−38:5)
TG(61:11)
PC(O−40:6)
SM(t31:2)
PC(37:4)
TG(O−54:7)
PC(O−32:1)
PC(39:4)
TG(46:2)
PC(35:1)
SM(d34:1)
PC(O−40:4)
TG(46:4)
TG(44:4)
TG(56:5)
PC(O−32:2)
PC(39:5)
PC(O−38:4)
PE(O−42:6)
TG(48:7)
PC(24:0)
TG(58:7)
TG(42:2)
PC(34:2)
SM(d35:1)
TG(56:7)
TG(57:4)
SM(d38:1)
TG(58:9)
TG(45:2)
TG(45:4)
TG(44:5)
LPA(18:2)
TG(43:0)
PE(O−42:5)
PC(O−36:4)
TG(55:2)
TG(62:12)
SM(d41:1)
TG(42:3)
PC(24:1)
TG(53:2)
TG(56:6)
TG(46:5)
PC(O−42:3)
PC(O−40:3)
SM(d39:1)
TG(58:8)
PE(O−38:6)
PC(O−41:5)
TG(60:12)
DG(O−34:0)
TG(O−54:5)
LPE(22:6)
PC(32:2)
PE(O−36:5)
PC(O−36:5)
PE(O−40:5)
SM(d40:1)
TG(60:11)
TG(46:6)
PC(36:2)
TG(54:10)
PC(33:0)
TG(44:2)
S1P(18:1)
SM(d36:1)
TG(43:2)
SM(d36:3)
PE(O−41:6)
LPE(16:0)
TG(55:4)
TG(46:1)
SM(d42:1)
SM(d44:1)
LPA(22:6)
TG(44:3)
SM(d43:2)
PE(34:0)
TG(59:10)
PA(34:2)
Cer(d42:0)
PC(44:3)
PE(O−40:6)
SM(d41:4)
TG(46:3)
PC(28:1)
PC(26:0)
PC(O−30:2)
TG(55:3)
TG(57:3)
LPC(24:0)
TG(48:6)
TG(55:5)
PC(O−36:6)
PC(32:0)
PC(O−44:4)
TG(O−56:8)
PE(O−40:7)
SM(d37:1)
TG(59:8)
TG(57:7)
LPE(18:0)
PC(38:3)
TG(53:3)
TG(62:14)
SM(d40:2)
TG(49:2)
TG(44:0)
PC(O−30:1)
PC(40:6)
TG(48:5)
TG(O−57:3)
TG(48:2)
PC(36:6)
PC(28:2)
LPA(20:4)
SM(d44:2)
TG(47:3)
RT_15.795
TG(51:2)
PC(O−32:0)
Cer(d38:2)
TG(55:6)
TG(47:5)
PC(O−44:9)
PE(O−40:3)
PC(O−42:4)
PE(O−40:8)
PE(O−39:5)
PC(O−41:4)
DG(O−36:3)
PC(34:4)
TG(47:2)
Cer(d46:5)
Cer(d42:1)
LPC(25:0)
PC(O−34:0)
TG(O−64:9)
LPA(18:1)
SM(d41:2)
PC(27:0)
TG(62:11)
SM(d28:0)
SM(t32:2)
TG(62:10)
PC(46:7)
LPA(16:0)
PC(O−44:3)
TG(O−64:8)
Cer(d40:0)
PE(O−42:7)
DG(O−34:2)
PC(44:7)
LPC(O−14:0)
SM(t34:1)
PE(30:0)
SM(t42:0)
SM(t41:1)
TG(64:12)
PE(O−44:6)
TG(52:10)
Cer(d44:1)
TG(O−56:7)
PC(O−34:1)
PC(O−44:10)
PC(O−43:5)
TG(45:3)
TG(60:9)
TG(43:3)
TG(O−58:6)
TG(O−59:3)
SM(t40:1)
SM(t31:1)
SM(d43:1)
TG(59:4)
TG(O−56:5)
DG(43:1)
PC(O−31:1)
DG(41:0)
SM(t36:1)
Cer(d41:0)
PE(37:4)
PS(38:4)
TG(O−58:7)
TG(O−50:0)
TG(48:4)
PE(O−42:8)
Cer(d36:0)
SM(d32:1)
PC(O−37:5)
PC(O−44:7)
LPC(22:0)
DG(28:1)
DG(O−38:5)
PC(O−31:0)
DG(39:0)
SM(d31:2)
SM(d38:2)
SM(d42:2)
PA(36:3)
LPC(23:0)
TG(45:1)
TG(57:5)
SM(d39:3)
PC(O−33:2)
PC(38:7)
PC(O−44:6)
SM(d34:3)
TG(47:1)
TG(45:5)
TG(48:3)
PE(O−38:3)
LPC(26:2)
PI(38:3)
PC(27:1)
SM(d42:4)
SM(d32:0)
SM(d30:0)
PC(O−39:6)
PE(O−38:5)
LPC(O−16:1)
PE(O−42:3)
PC(O−30:0)
PE(O−41:5)
TG(45:0)
SM(d41:3)
TG(O−56:4)
PE(40:6)
PC(O−36:1)
SM(d33:2)
PE(O−38:7)
TG(55:7)
PE(40:4)
PC(O−42:6)
SM(d34:2)
PC(37:5)
TG(57:8)
SM(t33:2)
PE(32:0)
PE(O−39:6)
SM(t33:0)
PC(42:8)
